# Reviews and Notices of Books, &c.

**Published:** 1869-02

**Authors:** 


### REVIEWS AND NOTICES OF BOOKS, &c.

We have just received from the house of LINDSAY & BLACKISTON (one of the largest book publishing houses of Philadelphia,) a *Theoretical and Practical Treatise* on MIDWIFERY, including the diseases of Pregnancy and Parturition—by P. CAZEAU, of Paris, member of the Imperial Academy of Medicine, etc.—revised and annotated by S. Tarnier, Adjunct Professor in the Faculty of Medicine, Paris, etc.; Fifth American from the Seventh French Edition, by Wm. R. Bullock, M. D., of Delaware.

This is a book of seven hundred pages, with 175 illustrations, printed on good paper with clear type. The mechanical execution and arrangement is in good taste. The work is one of undoubted merit, originally written by one of the most distinguished and learned French obstettricians, improved by the revision of an eminent Parisian accoucher, and translated into good English by Professor Bullock—making a volume worthy the confidence of the American profession.

We have also received from same medical publishers, No. 25 south 6th street, Philadelphia, BIDDLE'S MATERIA MEDICA, a work of nearly 400 pages. The illustrations, type, paper and mechanical execution is all that could be desired.

This work is more particularly intended for students. The author, John B. Biddle, M. D., is Professor of Materia Medica and Therapeutics in the Jefferson Medical College, and this the third edition of his work is much enlarged, and numerous articles not previously noticed have been added to the list of subjects presented. The *Hypodermic Method* of introducing medicines into the system, and the *atomization* or *pulverization* of fluids, are also treated of at length, as well as the various instruments employed for the purpose; making it not only valuable to the student, but to the practitioner.

Lindsay & Blackiston have also furnished us another valuable work of their publication. I refer to Hewitt on the Diseases of Woman—first American from the second London edition—revised and enlarged, containing 116 illustrations. Craily Hewitt, M. D., F. R. C. P., is Professor of Midwifery and Diseases of Woman in University College, and Obstetric Physician to University Hospital, London. In his work he includes the diagnosis, pathology and treatment of women, as well as the diagnosis of pregnancy. We think this a most valuable work, and worthy the attention of the profession. It is a book of 100 pages, printed in the best style of the art.

We have been favored with another work from the same gentlemanly publishers—a volume on Diseases of Children, by Thomas Hiller, M. D., of London. The original intention of the author was to publish the Clinical Lectures, as delivered by him at intervals, at the Hospital of Sick Children, Great Ormond street, London; but he abandoned the lecture style, and gives his experience derived from actual symptoms and treatment of the various diseases occuring in children from two to twelve years of age. He gives but little attention to surgical diseases or diseases of new-born children and infants under two years. The work contains nearly 400 pages of reading matter, with an appendix, in which we find many valuable formulæ for medicines in children's diseases. The work, so far as we have examined it, is well written, and should meet with general favor.

We are in receipt of still another work from Messrs. Lindsay & Blackiston, by S. B. Birch, M. D., member of the Royal College of Physicians of London, on the subject of Constipated Bowels : the various causes and the different means of cure. The author endeavors to take a rational view of a very common and very troublesome ailment. He could have selected no subject of greater interest to the American practitioner, and we doubt not but that this little work of 180 pages will reach the libraries of most of our reading men.

*Robertson on Extracting Teeth* is another little work which has just come to hand from the same publishers. It is a manual on extracting teeth, founded on the anatomy of the parts involved in the operation, the kinds and proper construction of the instruments to be used, the accidents liable to occur from the operation, and the proper remedies to retrieve such accidents: By Abraham Robertson, D. D. S., M. D., author of "Prize Essay on Extracting Teeth," etc. This is a work of 200 pages, and may be of great service to that part of our profession who are from necessity compelled to play the part of the dentist. The composition and mechanical execution of the work are both highly creditable, and for the interests and comfort of humanity we wish it a wide circulation.

We are also in receipt from the office of the Medical and Surgical Reporter a work of nearly 200 pages on the *Anatomy and Histology of the Human Eye*, by a A. Metz, M. D., Professor of Opthalmology in Charity Hospital Medical College, Cleveland, Ohio. This we consider a most valuable little work, and much needed by the profession.

We are in receipt of a little work of 120 pages from the publishing house of William Wood & Co., on *Spermatorrhœa its Causes, Symptomatology, Pathology, Diagnosis, Prognosis and Treatment*, by Robert Bartholow, A. M. M. D., Professor of Materia Medica and Therapeutics in the Medical College of Ohio, etc. Second edition revised and annotated. This is a work upon a subject to which too little attention has been given by the regular practitioner, and we trust it will meet with the success it merits.

